# Papillary thyroid cancer: the value of bilateral diagnostic lymphadenectomy

**DOI:** 10.1007/s00423-022-02493-w

**Published:** 2022-03-17

**Authors:** Jagdeep Singh Bhangu, Christoph Bichler, Julia Altmeier, Lindsay Hargitai, Andreas Selberherr, Peter Mazal, Jonas Brugger, Christian Scheuba, Philipp Riss, Bruno Niederle

**Affiliations:** 1 Division of General Surgery, Department of Surgery, Medical University of Vienna, Waehringer Guertel 18-20, 1090 Vienna, Austria; 2 Division of Clinical Pathology, Department of Pathology, Medical University of Vienna, Waehringer Guertel 18-20, 1090 Vienna, Austria; 3 Centre of Medical Statistics, Informatics and Intelligent Systems, Medical University of Vienna, Waehringer Guertel 18-20, 1090 Vienna, Austria

**Keywords:** Papillary thyroid carcinoma, Thyroidectomy, Lymph node dissection, Diagnostic lateral lymphadenectomy

## Abstract

**Purpose:**

Papillary thyroid carcinoma (PTC) spreads early to lymph nodes (LN). However, prophylactic central (CND) and lateral neck dissection (LND) is controversially discussed in patients with clinically negative nodes (cN0). The preoperative prediction of LN metastasis is desirable as re-operation is associated with higher morbidity and poor prognosis. The study aims to analyse possible benefits of a systemic bilateral diagnostic lateral lymphadenectomy (DLL) for intraoperative LN staging.

**Methods:**

Preoperative prediction of LN metastasis by conventional ultrasound (US) was correlated with the results of DLL and intra-/postoperative complications in 118 consecutive patients with PTC (cN0) undergoing initial thyroidectomy and bilateral CND and DLL.

**Results:**

Lateral LNs (pN1b) were positive in 43/118 (36.4%) patients, including skip lesions (*n* = 6; 14.0%). Preoperative US and intraoperative DLL suspected lateral LN metastasis in 19/236 (TP: 8.1%) and 54/236 (TP: 22.9%) sides at risk, which were confirmed by histology. Sixty-seven out of 236 (FN: 28.4%) and 32/236 (FN: 13.6%) sides at risk with negative preoperative US and intraoperative DLL lateral LN metastasis were documented. DLL was significantly superior compared to US regarding sensitivity (62.8% vs 22.1%; *p* < 0.002), positive predictive value (100% vs 76.0%), negative predictive value (82.4% vs 68.2%), and accuracy (86.4% vs 69.1%), but not specificity (100% vs 96.0%; *p* = 0.039). DLL-related complications (haematoma) occurred in 6/236 [2.5%] sides at risk, including chylous fistula in 2/118 [1.7%] patients.

**Conclusion:**

DLL can be recommended for LN staging during initial surgery in patients with PTC to detect occult lateral LN metastasis not suspected by US in order to plan lateral LN dissection.

## Introduction

Papillary thyroid carcinoma (PTC) is the most common endocrine tumour with a gradually increasing incidence over the past 20 years [1]. PTC accounts for almost 80% of all well-differentiated thyroid carcinomas [2]. At initial diagnosis, regional lymph node (LN) metastasis occurs in 30–80%, depending on diagnostic modalities [3]. While LN metastasis not removed during primary surgery and its role in recurrence is currently a subject of extended discussion [2, 4], recent studies have shown that metastasis in the lateral neck (levels 2 to 5 Robbins [5]; compartments C2/C3 Dralle [6]; staged as N1b according to the TNM classification) is, in addition to tumour size and extrathyroidal tumour infiltration, a further independent risk factor for negative prognosis [3, 7–9]. To date, the dissection of lateral LN ipsilateral or contralateral the primary tumour is only recommended in patients with clinically suspected or histologically proven lateral LN metastasis [2, 10].

Prophylactic central or lateral LN dissection is still a matter of discussion and is currently not recommended [11]. However, recent studies indicate that patients with lateral LN metastases are at higher risk for developing local recurrence [11, 12]. The preoperative prediction of LN metastasis in the central and lateral neck in their initial dissection would be valuable to avoid re-operations with higher morbidity and poor prognosis [3]. Furthermore, systematic LN dissection during initial surgery permits an accurate staging of disease, may decrease the rate of complications, guide a more appropriate subsequent treatment [8], and improve the prognosis of PTC.

The aim of this study was to evaluate the value of systematic extirpation of central jugular LNs (diagnostic lateral lymphadenectomy (DLL)) compared to the results of preoperative ultrasound (US) to predict uni- and/or bilateral lateral LN metastasis. Furthermore, the value of DLL for intraoperative LN staging for planning the extent of lateral LN dissection should be analysed.

## Patients and methods

### Demography

A total of 118 patients (females: 79/118 (66.9%); males: 39/118 (33.1%)), undergoing initial surgery for differentiated thyroid carcinoma at the Department of Surgery, Medical University of Vienna, were included in this analysis. Inclusion criteria were histologically confirmed PTC (clinically without LN metastasis [cN0]) and patients older than 18 years of age. Patients with follicular, anaplastic, or medullary thyroid cancer were excluded.

All patients with the pre- and intraoperative diagnosis of PTC were managed according to a prospective and standardized diagnostic, surgical, and pathohistological and follow-up protocol (standard operating procedure [SOP]).

The prospective data collection and the retrospective correlation analysis were approved by the Ethics Committee of the Medical University of Vienna (study protocol no. 1351/2017). All patients gave their written informed consent for all diagnostic and surgical procedures.

### Preoperative ultrasound

All patients underwent a preoperative neck US of the central and both lateral cervical regions from the skull down to the supraclavicular region (General Electric [GE] LOGIQ E9 device; 9 MHz probe). The left and right sides were documented separately. The US examinations were performed and analysed preoperatively by one radiologist. A suspicious LN was described with a solid content, sometimes necrotic or cystic, ovoid to round shape in the longitudinal plane but taller-than-wide in the transverse plane, hypoechogenic, with microcalcifications, irregular margins, loss of the nodal hilum, and peripheral vascularity [13].

### Surgery

Surgical treatment consisted of (total) thyroidectomy, initial bilateral central LN dissection (CND; extirpation of the lymphatic tissue along both recurrent laryngeal nerves without neuromonitoring [14] concerning Robbins level VI [5]; Dralle compartments C1a and C1b [6]), and bilateral diagnostic lateral lymph node extirpation (DLL = extirpation of the central jugular lymph nodes = lymphatic tissue lateral the internal jugular vein above and below the omohyoid muscle) [15] through a shortened Kocher’s skin incision (length dependent of the thyroid; between 30 and 40 mm). Independent of the preoperative US results, bilateral DLL was performed in all patients as part of the well-defined prospective SOP, as previous studies [15] have shown that positive LNs were histologically proven in 96% within this region independent of the size and the site of the primary thyroid tumour in patients with differentiated thyroid cancer and LN metastasis.

If LN metastasis was documented in lateral LNs by intraoperative frozen (DLL positive) section, an ipsilateral (if both DLL were positive: bilateral) functional lateral neck dissection (FLND) was performed. FLND was defined as complete removal of the lymphatic tissue from the base of the skull down to the upper thoracic outlet (Robins levels II to IV; Dralle C2 or C3) saving the strap muscles, the jugular vein, and all cervical nerves.

The results of US, intraoperative frozen sections of DLL, and the definitive histology were evaluated and correlated retrospectively with each other to assess the value of the methods to predict LN metastasis.

Intra- and postoperative complications of CND, DLL, and FLND were documented.

Following the SOP, postoperative treatment consisted of radioiodine ablation (80–100 mCi) 4 to 6 weeks after surgery. This allowed to document persisting disease in the lateral neck or distant metastasis at the time of surgery.

All patients were monitored annually as outpatients in accordance with a standardized follow-up protocol that included clinical examination, ultrasonography of the neck, biochemical measurements of thyroglobulin levels, and radiography of the lungs. However, long-term follow-up results were not the subject of this presentation [16, 17].

Concerning the extent of LN dissection, patients were assigned into three groups: group 1: patients with bilateral CND and negative bilateral DLL frozen sections (*n* = 77 patients); group 2: patients with bilateral CND and bilateral DLL and unilateral positive frozen sections, uni-/ipsilateral FLND (*n* = 28); and group 3: patients with bilateral CND and bilateral DLL and bilateral positive frozen sections on both FLND (*n* = 13).

### Statistical analyses

CND and DLL were performed in 118 patients on both sides. Therefore, 236 “nerves at risk” and 236 “sides at risk” were used for the statistical calculation.

All statistical analyses were conducted in SPSS Statistics 25.0 software (SPSS Inc., Chicago, IL, USA). We compared the results of preoperative US, DLL (frozen section), and definitive histology among each other using McNemar’s test. The sensitivity, specificity, positive predictive value (PPV), negative predictive value (NPV), and the general accuracy were determined for US and DLL and subsequently compared with a two-proportion *Z*-test. The significance level was adjusted using the Bonferroni method (*p* ≤ 0.01).

## Results

### TNM classification

According to the current UICC/TNM Classification 15, 62 (52.5%) tumours were identified as pT1 (tumour diameter: ≤ 20 mm [35 (56.5%) subclassified as pT1a (≤ 10 mm); 27 (43.5%) as pT1b (11–20 mm)]). Twenty-three patients (19.5%) had a pT2 tumour (21–40 mm) and 20 patients (17.0%) a pT3 tumour (> 40 mm). In 13 (11.0%) patients, the carcinoma had already grossly invaded the subcutaneous fatty tissue, larynx, trachea, oesophagus, recurrent laryngeal nerve, prevertebral fascia, and carotid artery (pT4 [8 (61.5%) subclassified as pT4a; 5 (38.5%) as pT4b]) (see Table 1).

**Table 1:** TNM classification [18] of 118 patients with papillary thyroid cancer (PTC)

Classification						
T	*n*	N0, *n* (%)	N1a, *n* (%)	N1b, *n* (%)	M0, *n* (%)	M1, *n* (%)
pT1	62	30 (48.4)	17 (27.4)	15 (24.2)	62 (100)	0
pT1a	35	21 (60.0)	7 (20.0)	7 (20.0)	35 (100)	0
pT1b	27	9 (33.3)	10 (37.0)	8 (29.6)	27 (100)	0
pT2	23	7 (30.4)	8 (34.8)	8 (34.8)	23 (100)	0
pT3	20	7 (35.0)	3 (15.0)	10 (50.0)	18 (90.0)	2 (10.0)
pT4	13	2 (15.4)	1 (7.7)	10 (76.9)	12 (92.3)	1 (7.7)
pT4a	8	2 (25.0)	0	6 (75.0)	8 (100%)	0
pT4b	5	0	1 (20.0)	4 (80.0)	5 (100%)	0
Total	118	46 (39.0)	29 (24.6)	43* (36.4)	115 (97.5)	3 (2.5)

TNM classification—UICC 8th edition

*T*, tumour; *LN*, lymph node; *N0*, no LN metastasis; *N1a*, central LN; *N1b*, lateral/bilateral LN metastasis; *M0*, no distant metastasis; *M1*, distant metastasis; *p*, pathological classification—pT1a: ≤ 10 mm and intrathyroidal; pT1b: 11–20 mm and intrathyroidal; pT2: 21–40 mm and intrathyroidal; pT3: > 40 mm and intrathyroidal or gross extrathyroidal extension; pT4a: any size and gross extrathyroidal extension; pT4b: gross extrathyroidal extension or encasing the carotid artery, mediastinal vessels

^*^Six out of 43 (14.0%) patients with LN metastasis had skip lesions

In 32 patients (27.1%), PTC was localized in the left thyroid lobe, in 41 patients (34.8%) in the right thyroid lobe, and in six cases (5.1%) in the area of the isthmus.

Unilateral multicentricity was identified in 14 patients (11.9% [group 1 (*n* = 7), group 2 (*n* = 6), group 3 (*n* = 1)]) and bilateral multicentricity in 39 patients (33.1% [group 1 (*n* = 26), group 2 (*n* = 6), group 3 (*n* = 7)]).

The median size of PTC was 20 mm (range 2–70 mm; group 1: median 15 mm (range 2–70 mm), group 2: 23 mm (range 2–70 mm), group 3: 45 mm (range 5–60 mm)).

### LN metastasis

In 46/118 patients (39.0%), neither central nor DLL revealed LN metastasis (pN0). In 72/118 patients (61.0%), PTC had metastasized to the cervical LNs (pN1). Twenty-nine (40.3%) of these patients showed central LN only (pN1a) and 43 patients (59.7%) lateral LN involvement (pN1b). Six patients (6/43 [14.0%] cases) with lateral LNs presented with skip lesions.

Median 9 (range 0–45) and 10 (range 0–49) LNs were removed from the left and right neck compartments, respectively, via DLL.

Thirty out of 118 (25.4%) patients with pT1 tumour (pT1a: 21/30 (70.0%); pT1b: 9/30 (30.0%)) were classified pN0. Seventeen patients (27.4%) with pT1 tumour presented with central LN metastases (pT1a: 7/20 (20.0%) and pT1b: 10/27 (37.0%)) and 15 patients (24.2%) lateral LN metastases (pT1a: 7/35 (20.0%); pT1b: 8/27 (29.6%)). Three out of 15 (20.0%) patients with pT1N1b tumours showed skip lesions, two of them with multifocal microcarcinomas (pT1am) and one with pT1b tumour.

Furthermore, LN metastases were absent in patients with 7/23 (30.4%) pT2 and in 7/20 (35.0%) pT3 (*n* = 7) and 2/13 (15.4%) pT4 tumours. PTC smaller than 40 mm and limited to the thyroid gland (pT2) equally infiltrated the central (pN1a: 34.8%, *n* = 8) and lateral LNs (pN1b: 34.8%, *n* = 8), while pT3 carcinomas metastasized to the lateral cervical compartment more frequently (pN1b: 50.0%, *n* = 10) than to the central cervical compartment (pN1a: 15.0%, *n* = 3). Tumours with extension into adjacent neck structures (pT4) showed metastasis in the central neck compartment in 1/13 (7.7%) patient and in the lateral LNs in 10/13 (76.9%) patients, respectively.

### Distant metastasis

According to the postoperative radioiodine ablation, whole-body scan distant metastases occurred in three patients (2.5%; 2/20 [10.0%] patients with pT3 and 1/13 [7.7%] patient with a pT4 tumour). Distant metastasis was not observed in patients with pT1 and pT2 tumours.

### Preoperative ultrasonography

Ultrasonography (US) predicted positive lateral LN in 25/236 (10.6%) and was negative in 211/236 (89.4%) examinations, respectively. In 19/236 (8.1%) US scans, lateral LN metastases were histologically confirmed (true positive [TP]) and 144/236 (61.1%) scans were true negative (TN). US was unable to detect LN metastases in 67/236 (28.4%) examinations (false negative [FN]) and incorrectly predicted LN metastasis in 6/236 (2.5%) scans (false positive [FP]). The sensitivity of US was 22.1%, specificity 96.0%, PPV 76.0%, NPV 68.2%, and accuracy 69.1%. Data is summarized in Table 2.

**Table 2:** Results of bilateral ultrasound (US) and bilateral diagnostic lateral lymphadenectomy (DLL) in patients

LN metastasis	US	95% CI	DLL	95% CI	*p* value
True positive, *n* (%)	19/236 (8.1)		54/236 (22.9)		< 0.001
True negative, *n* (%)	144/236 (61.0)		150/236 (63.6)		0.635
False positive, *n* (%)	6/236 (2.5)		0		0.040
False negative, *n* (%)	67/236 (28.4)		32/236 (13.6)		< 0.001
Sensitivity	22.1%	0.146–0.319	62.8%	0.522–0.723	< 0.001
Specificity	96.0%	0.915–0.982	100%	0.975–1.000	0.039
PPV	76.0%	0.566–0.885	100%	0.934–1.000	0.001
NPV	68.2%	0.617–0.742	82.4%	0.762–0.873	0.002
Accuracy	69.1%	0.629–0.746	86.4%	0.815–0.902	< 0.001

*LN*, lymph node; *CI*, confidence interval; *PPV*, positive predictive value; *NPV*, negative predictive value

### DLL and frozen section

The results of 54/236 (22.9%) positive (TP) and 150/236 (63.6%) negative frozen section samples (TN) were subsequently confirmed by definitive histological examination. In 32/236 (13.6%) DLLs, the result of frozen section was negative. However, LN metastases were found in the definitive histological examination (FN). No false-positive frozen section results were found in this analysis. The DLL and frozen section analysis displayed a sensitivity of 62.8% and a specificity of 100%. PPV was 100% and NPV was 82.4%. The accuracy was 86.4% (Table 2).

### Comparison of US and DLL

US and DLL showed a significant difference in terms of sensitivity (*p* < 0.001), PPV (*p* = 0.001), NPV (*p* = 0.002), and accuracy (*p* ≤ 0.001) but did not differ in regard to specificity (*p* = 0.039) (Table 2).

### FLND

Unilateral and bilateral FLNDs were conducted in 28/236 (11.9%) and 26/236 (11.0%) cases. Fifty percent (14/28) of the unilateral and 65.4% (17/26) of the bilateral FLNDs were positive in the definitive histological examination. Only 1/13 (7.7%) patient, who underwent bilateral FLND (1/26 [3.8%]) and showed no LN metastases in the definitive histological examination, presented a postoperative complication (haematoma). During unilateral and bilateral FLNDs, medians of 21 (range 2–67) and 29 (range 9–87) LNs were removed.

### Postoperative complications

In total, 15 patients (12.7%) showed complications after surgery.

After thyroidectomy and bilateral CND, unilateral paralysis of the recurrent laryngeal nerve (RLN) was documented in 7/118 (5.9%) patients (7/236 [3.0%] nerves at risk; unilateral temporary paralysis of the RLN: 6/236 [2.5%] nerves at risk; unilateral permanent paralysis of the RLN: 1/236 [0.4%] nerve at risk). Combined with the FLND, no nerve injuries to the accessory, hypoglossal, or vagal nerves, to branches of the cervical or brachial plexus, or to the sympathetic trunk were observed.

After DLL, postoperative bleeding or haematoma with surgical re-intervention were reported in 4/118 (3.4%) patients (4/236 [1.7%] DLLs). Chylous fistula appeared following 2/118 (1.7%) DLLs on postoperative days 1 and 7. In the first patient, the thoracic duct showed an anatomical variety on its course to the internal jugular vein. The thoracic duct proceeded dorsally of the common carotid artery to the middle of the neck, crossed the vagal nerve and the internal jugular vein moving parallel down to the venous angle of internal jugular and left subclavian vein. In the second case, the thoracic duct was injured due to preparation of lymphatic tissue near the upper thoracic outlet. In both cases, the fistulas were occluded surgically plugging the leak with parts of the omohyoid muscle. Both patients were discharged 2 days after re-intervention.

After FLND, postoperative bleeding or haematoma with surgical re-intervention occurred in 2/118 (1.7%) patients (1/28 [3.6%] unilateral FLND; 1/26 [3.8%] bilateral FLND; Table 3).

**Table 3:** Postoperative complications

	CND, *n* (%)	DLL, *n* (%)	FLND unilateral, *n* (%)	FLND bilateral, *n* (%)
Patients	118 (100)	118 (100)	28/118 (23.7)	13/118 (11.0)
RLN/sides at risk*	236 (100)	236 (100)		
No complications	229/236 (97.0)	230/236 (97.5)	27/28 (96.4)	25/26 (96.2)
Overall local complications	7/236 (3.0)	6/236 (2.5)	1/28 (3.6)	1/26 (3.8)
Temporary unilateral VFP	6/236 (2.5)	–	–	–
Permanent unilateral VFP	1/236 (0.4)	–	–	–
Haematoma	–	4/236 (1.7)	1/28 (3.6)	1/26 (3.8)
Chylous fistula—neck	–	2/118 (1.7)	0/118	0/118

*CND*, central neck dissection; *DLL*, diagnostic lateral lymphadenectomy; *FLND*, functional lateral neck dissection; *VFP*, vocal fold paralysis; *RLN*, recurrent laryngeal nerve

^*^Nerves/sides at risk: *n* = 236

Neither clinically nor sonographically lateral LN recurrences were documented during a 5-year follow-up in the 118 study patients.

## Discussion

In the current study, 29/118 (24.6%) patients with central (pN1a) and 43/118 (36.4%) patients with lateral (pN1b) metastases, including 6/118 (5.1%) patients with “skip lesions”, were documented independent of the pT classification. Preoperatively, all patients were cN0. Intraoperative frozen sections and definitive histological reports of diagnostic bilateral DLL were correlated with the findings of preoperative US. An accurate preoperative evaluation of the central and lateral compartment is needed to locate suspicious lymph nodes, given that prophylactic LN dissection is recommended in patients with a high risk of LN metastasis [19].

US is a non-invasive diagnostic modality that is recommended in the preoperative work-up of thyroid lesions and detection of abnormal LNs in the lateral compartment. However, varying sensitivity (37 to 93%) and specificity rates (79 to 100%) have been reported in the literature and may depend on the size and number of affected nodes [20–22]. The current results demonstrate a lower sensitivity (22.1%), specificity (96.0%), PPV (76.0%), and NPV (68.2%), indicating a weakness of US in detecting lateral LN metastasis. Other authors presented similar sensitivity and specificity rates for US diagnosing lateral LN metastasis [23].

In this study, DLL demonstrates a sensitivity of 62.8%, specificity 100%, PPV 100%, NPV 82.4%, and accuracy 86.4% in identifying lateral LN metastasis. Compared to preoperative US, DLL presents significantly superior results. Notably, 27/98 (27.6%) patients in this study with negative preoperative US presented with histological LN metastasis in the lateral compartment, emphasizing the importance of DLL in thyroid cancer surgery. When taking the TP and TN rates into account, DLL influenced the extent of lateral neck surgery in 86.5% of the patients. Therefore, the main advantage of DLL lies in its ability to stage lateral LN metastasis not predicted by preoperative US. Only DLL and intraoperative frozen sections allow the planning of adequate lateral LN surgery.

In PTC, prophylactic bilateral CND can lead to complete remission of the disease in the central neck in patients with clinically occult LN metastasis, reducing the chances of recurrence and subsequent morbidity of re-operations. It also allows a definitive LN staging to plan further adjuvant therapy. CND can be performed with an overall low morbidity [24]. Patients with metastasis in the lateral neck show a poorer prognosis compared to those with metastasis in the central neck or those with no metastasis at all [1, 25]. Functional dissection in patients with documented lateral LN metastasis improves survival, given that lateral LN metastasis negatively influences overall survival [26, 27].

The knowledge of the pattern of cervical nodal metastasis is of great importance, as the central compartment (prelaryngeal, pre-/paratracheal, upper mediastinum) is known to be the first site of lymphatic spread in the majority of patients, metastasizing next to the ipsilateral lateral compartment [28]. Generally, the caudal portion of the lateral compartment is affected more frequently than the cranial portion [29, 30], implicating that the cervical lymphatic flow is directed toward the ipsilateral lower jugular LNs and the venous angle. In this study, 72/118 patients (61.0%; pT1–pT4) were identified in whom the PTC had metastasized to the cervical LNs (pN1). The current findings emphasize the importance of CND in the initial surgery, given that the predictive value of US in detecting LN metastases in the central compartment is very low [20, 31].

Interestingly, some patients present with metastasis in the lateral compartment without involving the central compartment (“skip lesions”) [32]. Forty-three of 72 patients (59.7%) showed lateral LN involvement (pN1b), including six patients (14.0%) with skip lesions. This rate lies within the range of 4 to 38% of patients documented in the literature [8, 33]. This finding is quite relevant, given that in the absence of DLL, these patients would have been falsely classified as pN0 [34, 35]. DLL documented clinically occult “skip lesions” in all patients.

In terms of the surgical strategy applied in this study, CND and bilateral DLL are always performed prophylactically [15], followed by FLND only in the case that positive lateral LN metastasis is diagnosed by frozen section during initial surgery for PTC [16, 17]. CND demonstrates an overall low rate of morbidity and is recommended in various international guidelines [24]. Bilateral DLL is performed using the typical transverse skin incision without additional extension and poses no additional risk when conducted in combination with initial thyroidectomy and CND. Moreover, DLL and the subsequent frozen section examination are important tools in the intraoperative staging of PTC. In the case of lateral LN involvement, the majority of LN metastasis are located above and below the omohyoid muscle corresponding to the caudal parts of LN level 3 and cranial parts of LN level 4 [36].

A therapeutic LN dissection is the fundamental treatment for clinically evident cervical LN metastasis. Cervical LN dissection range from a “berry-picking” approach, in which only grossly involved”, enlarged” LNs are excised, to “systematic” en bloc compartment-oriented LN dissection, where one or more LN levels (compartments) are removed. Today, the “berry-picking” approach has become obsolete, given that in patients with clinically palpable LNs, the tumour has always metastasized into smaller LNs, thereby predisposing these patients to recurrent cancer [30, 37]. Furthermore, systematic compartment-oriented procedures show a significant less local recurrence rate compared to the “berry-picking” procedure. There is no definitive evidence of decreased morbidity after applying this “selective” approach [38]. A “berry-picking approach” is only recommended in cases of local LN recurrence preceding systematic LN dissection or CND [32].

In the past decade, proposals for the establishment of a diagnostic pathway similar to the sentinel LN mapping (SLNM) in breast cancer, cutaneous melanoma, and other malignant solid neoplasms in PTC have emerged. The concept of SLNM is a less invasive method to evaluate metastasis in clinically normal LN staging and indicates LN dissection only in patients with histologically documented SLNM. The thyroid gland possesses a rich lymphatic drainage system. There is an initial horizontal (central) and vertical (lateral) lymphatic way of metastatic formation in cervical LN metastasis. Both ways develop in parallel and the prediction of SLNM in the central or lateral neck seems difficult and varies individually. Up to 70% of patients demonstrate a SLN in more than one location. By definition [32], skip lesions were revealed as positive SLNM in 14% in the current analysis. Studies have reported an accuracy of SLNM in detecting LN in the central compartment in up to 97%, though only up to 33% in the lateral compartment [39].

The presence of cervical LN metastasis seems to be the most significant prognostic factor for patients with PTC. Locoregional recurrences tend to occur more often in patients with LN metastases and result in poor prognosis and decreased survival. Studies have indicated an age > 45 and an increasing primary tumour size as further independent factors predicting worse prognosis, whereas gender, multifocality, and operative strategy did not reach prognostic significance [16, 17, 40, 41].

Respecting the results of TP and TN rates, DLL influenced the extent of lateral neck surgery in 86.5% of the patients. Therefore, the main benefit of DLL is the possibility to stage lateral LN metastasis not predicted by preoperative US. Only DLL and intraoperative frozen sections allow the planning of an adequate lateral LN surgery.

DLL did not significantly increase the risk for postoperative complications when performed in addition to initial (total) thyroidectomy and CND. In the current study, the overall complication rate was 2.5%, including one patient with local haematoma and two others with a chylous fistula (2/118 [1.7%]). The reason for these uncharacteristic complications was, on the one hand, an anatomical variety of the thoracic duct, and, on the other hand, a dissection of the lymphatic tissue too close to the confluence of the thoracic duct into the internal jugular vein in the upper thoracic outlet (not the typical region of DDL). The risk for such complications can be minimized with an even more meticulous preparation. Other authors have reported a similar complication rate in patients undergoing thyroidectomy in combination with CND, indicating that DLL does not necessarily increase the risk for postoperative compilations [42]. Furthermore, DLL does not cause any adverse long-term consequences or negative postoperative oncological impairments. Using the common transverse Kocher’s skin incision (the length adapted to the size of the largest thyroid nodule) without additional extension, the postoperative cosmetic result remains the same as after thyroidectomy with CND only.

This retrospective analysis has a few limitations. Firstly, only a small patient population is investigated which does not respect the existence of micrometastasis as well as the size and number of affected LNs in detail especially in patients with false-negative DLL. Secondly, this study does not address the issue of possible occurrence of long-term recurrence. The focus of this study was on evaluating the benefits of DLL in detecting occult LN metastasis. Thus, an independent assessment of these important matters is necessary in a prospective randomized study.

## Conclusions

Considering the large number of clinically occult (cN0) and ultrasonographically negative LN metastasis in the lateral neck and the metastatic involvement of LNs caudal in level 3/cranial in level 4 (= ventral jugular) being representative of the involvement of the lateral neck, DLL appears to be a useful procedure in planning the extent of LN surgery in patients with PTC.
